# Levodopa Changes Functional Connectivity Patterns in Subregions of the Primary Motor Cortex in Patients With Parkinson’s Disease

**DOI:** 10.3389/fnins.2020.00647

**Published:** 2020-07-08

**Authors:** Yang Shen, Jun Hu, Yong Chen, Wan Liu, Yuqian Li, Lei Yan, Chunming Xie, Wenbin Zhang, Miao Yu, Weiguo Liu

**Affiliations:** ^1^Department of Neurology, The Affiliated Brain Hospital of Nanjing Medical University, Nanjing, China; ^2^Department of Radiology, The Affiliated Brain Hospital of Nanjing Medical University, Nanjing, China; ^3^Department of Laboratory Medicine, The Affiliated Brain Hospital of Nanjing Medical University, Nanjing, China; ^4^Department of Rehabilitation, The Affiliated Brain Hospital of Nanjing Medical University, Nanjing, China; ^5^Department of Neurology, Affiliated ZhongDa Hospital, School of Medicine, Southeast University, Nanjing, China; ^6^Department of Functional Neurosurgery, The Affiliated Brain Hospital of Nanjing Medical University, Nanjing, China

**Keywords:** Parkinson’s disease, primary motor cortex, subregion, acute levodopa challenge test, resting-state functional magnetic resonance imaging

## Abstract

**Background:**

The primary motor cortex (M1) is a critical node in Parkinson’s disease (PD)-related motor circuitry; however, the functional roles of its subregions are poorly understood. In this study, we investigated changes in the functional connectivity patterns of M1 subregions and their relationships to improved clinical symptoms following levodopa administration.

**Methods:**

Thirty-six PD patients and 37 healthy controls (HCs) were enrolled. A formal levodopa challenge test was conducted in the PD group, and the Unified Parkinson’s Disease Rating Scale motor section (UPDRS-III) was assessed before (off state) and 1 h after administration of levodopa (on state). The PD group underwent resting-state functional magnetic resonance imaging in both off and on states, whereas the HC group was scanned once. We used the Human Brainnetome Atlas template to subdivide M1 into twelve regions of interest (ROIs). Functional connectivity (FC) was compared between PD on and off states [paired *t*-test, voxel-level *p* < 0.001, cluster-level *p* < 0.05, Gaussian random field (GRF) correction] and between patients and HC (two-sample *t*-test voxel-level *p* < 0.001, cluster-level *p* < 0.05). Correlations between ΔFC (differences in FC between PD off and on states) and clinical symptom improvements were examined.

**Results:**

There was decreased FC between the right caudal dorsolateral area 6 and the anterior cingulate gyrus (ACC), the right upper limb region and the left medial dorsal thalamus (mdTHA), as well as increased FC between the left tongue and larynx region and the left medial frontal gyrus. ΔFC between the right caudal dorsolateral area 6 and ACC was positively correlated with improvements in UPDRS-III total scores as well as the rigidity (item 22) and bradykinesia (items 23–26 and 31) subscores. ΔFC between the right upper limb region and left thalamus was positively correlated with improvements in the left upper limb tremor (items 20c and 21b) and postural tremor (item 21b) subscores.

**Conclusions:**

Our results reveal novel information regarding the underlying mechanisms in the motor circuits in the M1 and a promising way to explore the internal function of the M1 in PD patients. Notably, M1 is a potential therapeutic target in PD, and the exploration of its subregions provides a basis and a source of new insights for clinical intervention and precise drug treatment.

## Introduction

Parkinson’s disease (PD) is a progressive neurodegenerative disorder, and the underlying mechanism of its pathophysiology is the degeneration of dopaminergic neurons in the substantia nigra, which leads to dopamine depletion in the striatum. This depletion causes dysfunction within the basal ganglia-thalamus-motor cortex (BGMC) circuit, resulting in progressive motor symptoms including resting tremor, bradykinesia, rigidity, and postural instability (Wu and Hallett, 2005; Lees et al., 2009; Akram et al., 2017). Previous neuroimaging studies have suggested that levodopa treatment could partially normalize the abnormal functional connectivity (FC) in the BGMC circuit in PD patients. In addition, altered FC showed a significant correlation with improvements in clinical symptoms after levodopa intervention (Wu et al., 2009b, 2011; Agosta et al., 2014; Gao et al., 2017).

One of the critical nodes in this motor circuit is the primary motor cortex (M1), which plays a key role in generating neural impulses that regulate movements (Burciu and Vaillancourt, 2018; Hensel et al., 2019). The motor cortex (MC) includes M1 that not only participates in the classic BGMC circuit, which is used to explain the underlying mechanism of bradykinesia and rigidity, (DeLong and Wichmann, 2015) but also participates in the MC-cerebellum-thalamus circuit, which could explain resting tremor (Helmich et al., 2011; Dirkx et al., 2017). For instance, a resting-state functional magnetic resonance imaging (rs-fMRI) study found that PD patients with leading symptoms of akinesia showed a significantly stronger connectivity between the right M1 and pre-supplemental motor area (SMA) than normal subjects, and altered FC was positively correlated with improvements in motor scores (Wu et al., 2011). In another study, FC between the M1 and ventral intermediate nucleus (VIM) of the thalamus was increased in tremor-dominant PD patients and showed a positive correlation with clinical resting tremor scores (Zhang et al., 2016). Furthermore, Helmich et al. (2011) found that tremor-dominant PD patients exhibited increased functional coupling between the internal globus pallidus (GPi)/putamen and the MC compared to matched non-tremor PD patients and healthy controls. This increased functional coupling showed a significant positive correlation with clinical resting tremor scores (Helmich et al., 2011). Thus, the functional activity of the MC, particularly the M1, is closely related to clinical motor symptoms in PD patients.

Simultaneously, a variety of other neuroimaging techniques have further focused on the functional activity of M1 in PD patients. In task-based fMRI studies, reduced functional activity in M1 has been reported in both drug-naïve PD and PD patients tested off dopaminergic medication, (Buhmann et al., 2003; Spraker et al., 2010; Burciu et al., 2015; Mohl et al., 2017) whereas increased activation in M1 has also been reported in PD patients receiving dopaminergic treatment (Haslinger et al., 2001). In addition, Holtbernd et al. (2014) suggested in a positron emission tomography (PET) study that abnormal metabolism in M1 could occur even in idiopathic rapid-eye-movement sleep behavior disorder (RBD), which is currently considered to be a precursor of PD (Holtbernd et al., 2014). Accordingly, a consistent conclusion is that in the resting state and during motor tasks, the functional activity of M1 is altered throughout all stages of PD relative to healthy controls (Prodoehl et al., 2010; Mohl et al., 2017).

However, there are still inconsistent views on M1 in PD-related studies. One of the contradictory views is that the regional homogeneity (ReHo) across the basal ganglia as well as M1 is reduced in PD patients and further declines with disease progression (Pan et al., 2017; Zeng et al., 2017). Nevertheless, another PD study showed an increased ReHo in the M1, which can be normalized by levodopa (Wu et al., 2009a). This inconsistent result may be partly due to differences in the enrolled patient’s age, disease duration, and dominant symptoms. More importantly, this could be attributed to the fact that M1 contains a wide range of areas and complex internal functions. Penfield and Rasmussen (1950) drew the motor homunculus, which proposed that different regions in M1 may have different functions. However, this map also contained overlaps, reversals, and fractures (Graziano, 2016). Furthermore, Graziano (2016) found that the MC appeared to contain functional zones, each of which may emphasize a complex, ethologically meaningful category of behavior (Graziano, 2016). It is worth mentioning that most previous PD-related studies have examined the M1 as a whole rather than exploring the potential differential functions of its subregions. In fact, whether subregions of the M1 exhibit different changes in connection patterns after drug intervention in PD patients, as an exploratory study, warrants further attention.

Currently, levodopa is still the most efficacious and essential therapeutic drug for PD. The short half-life of levodopa and the dependence of striatal dopamine synthesis upon external levodopa in PD means that levodopa administration can immediately translate into clinical effects (Nutt, 2008; Politi et al., 2018). However, long-term, chronic levodopa treatment potentially introduces many confounds, such as differences across patients in daily frequency of administration and doses of levodopa. Therefore, to avoid long-term effects, an acute oral levodopa challenge test is widely used in PD studies to observe the effects of levodopa on clinical symptoms and motor circuits. As a timesaving and quick readout tool, the acute levodopa challenge test could improve the accuracy of clinical differential diagnoses (Schade et al., 2017). In addition, a good response to a levodopa challenge test is an important predictor of favorable long-term outcomes, especially for the preoperative evaluation of deep brain stimulation (DBS) (Machado et al., 2006). Finally, a formal levodopa challenge test could also avoid the bias of other anti-Parkinson’s drug interventions, such as dopamine agonists.

In the present study, we used the Brainnetome Atlas template (Fan et al., 2016) to divide M1 into 12 subregions and explored the changes in FC in each subregion before (off state) and after (on state) an acute levodopa challenge test in PD patients. The Brainnetome Atlas template utilized the differences in the structural connection pattern of each voxel [diffusion tensor imaging (DTI) for fiber tracking] and aggregated the voxels with the same connection patterns by a clustering algorithm to complete the definition of brain region boundary (Fan et al., 2016; Paxinos, 2016). In other studies, this template has provided a more detailed understanding of the differences in functional connectivity in different subregions of brain areas, such as the thalamus (Li et al., 2019) and Broca’s area (Zhang et al., 2017). Rs-fMRI is a non-invasive method to investigate brain activity and neural network connectivity (Fox and Greicius, 2010; Prodoehl et al., 2014). More importantly, unlike the brain’s structural networks, functional networks are considered to capture the dynamics of information communication among different regions (Yu et al., 2017). Therefore, we observed the dynamic changes in FC in each subregion of the M1 in PD and hypothesized that (1) there are differential changes in connection patterns in the subregions of the M1 after levodopa intervention and (2) the changed connectivity patterns are correlated with improvements in specific clinical symptoms.

## Materials and Methods

### Subjects

Fifty-five PD patients and 37 healthy controls (HCs) were recruited from the Affiliated Brain Hospital of Nanjing Medical University. PD patients were diagnosed by an experienced neurologist according to the United Kingdom Parkinson’s Disease Society Brain Bank Clinical Diagnostic Criteria (Hughes et al., 1992). All PD patients and HCs underwent a structural brain MRI to rule out dementia and significant brain atrophy. The requirements for all subjects were as follows: (1) right-handed, (2) aged between 50 and 75 years old, and (3) vision or corrected vision and binaural hearing could meet the needs of the evaluation and could be used to complete the examination. The inclusion criteria for the PD patients included the following: (1) the diagnosis of PD fulfilled the United Kingdom Parkinson Disease Society Brain Bank Criteria for idiopathic PD, (2) the course of the disease was more than 1 year, (3) anti-PD drugs were stable in the previous 3 months, and (4) Mini-Mental State Examination (MMSE) score ≥ 24. The exclusion criteria for all subjects were as follows: (1) history of disturbance of consciousness, (2) history of familial inherited diseases, (3) history of schizophrenia, manic episodes, or other mental diseases, (4) history of alcohol or drug dependence, (5) complications with severe heart, liver, kidney, brain, and hematopoietic system diseases, (6) contraindications for MRI scanning such as electronic and metal appliance implantation, and (7) T2-weighted MRI showing cerebral infarction or vascular injury.

### Study Procedure

**FIGURE 1:**
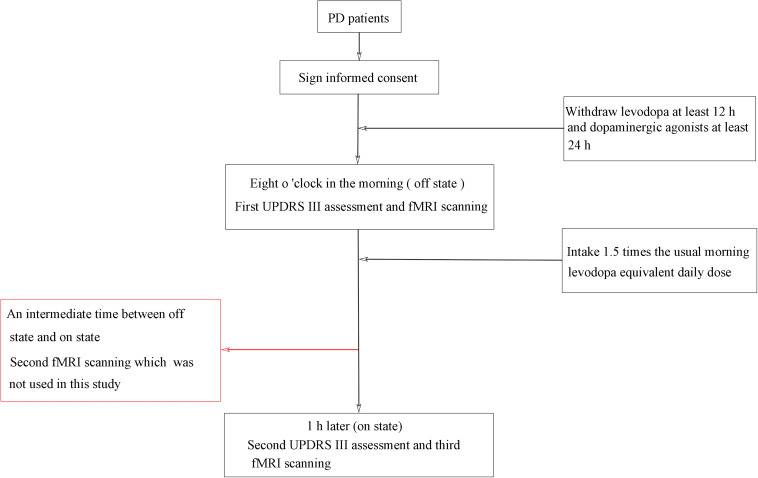
Flowchart of the study procedure.

A standardized, acute levodopa challenge test (Moreau et al., 2015; Fabbri et al., 2017) was conducted in the fasting state and in both off and on levodopa conditions (see Figure 1), and the Unified Parkinson’s Disease Rating Scale (UPDRS) (Vassar et al., 2012) was assessed by an experienced neurologist. PD patients were assessed in the off state (withdrawing levodopa at least 12 h and dopaminergic agonists at least 24 h) and assessed again 1 h or when in a clinically on state after the patients took 1.5 times the usual morning levodopa equivalent daily dose (Lopiano et al., 2002; Lang et al., 2006; Rodriguez et al., 2007; Rabel et al., 2016). All patients showed a positive response to levodopa, as indicated by reduced motor symptoms. PD patients were scanned three times (“off” state, “on” state, and an intermediate time between “off” states and “on” states), whereas healthy control subjects were scanned only once. In this study, we included data for only two scanning times in the PD patients: off state and on state. The patients were also assessed on the H and Y staging scale (Hoehn and Yahr, 1967) and the MMSE (Folstein et al., 1975) while on their medication.

For this study, we selected 39 PD patients with more than 30% improvement of UPDRS-III (motor section). Two PD patients were excluded because of poor MR image quality, and one PD patient was excluded as an outlier in terms of unusually large head motion (see below). The demographics and clinical details of the remaining 36 PD patients (61.0 ± 4.6 years; 19 M/17 F) and 37 control subjects (59.7 ± 4.3 years; 19 M/18 F) are shown in Tables 1, 2. All assessments were performed in accordance with the Declaration of Helsinki, and the protocol was approved by the Medical Ethics Committee of the Affiliated Brain Hospital of Nanjing Medical University. All subjects provided written informed consent for participation in the study.

**TABLE 1:** Demographic and clinical characteristics of the sample (mean ± SD).

Groups	PD	HC	*t*/χ^2^	*p-*value
Age (years)	61.0 ± 4.6	59.7 ± 4.3	1.26	0.211
Sex (female/male)	17/19	18/19	0.02	0.903
Education	12.0 ± 3.67	11.6 ± 4.7	0.41	0.681
MMSE	28.4 ± 1.6	29.0 ± 1.5	−1.61	0.112
Disease duration (years)	6.3 ± 4.3	NA		
H and Y stage	2.2 ± 0.7	NA		
LED dose (mg)	455.2 ± 232.4	NA		

*Group differences in age, education, and MMSE using two-sample t-test. Sex distribution assessed by chi-squared test. MMSE, Mini-Mental State Exam; H and Y, Hoehn and Yahr staging; LED, levodopa equivalent daily dose. NA, not available*.

**TABLE 2:** Clinical characteristics of the sample before and after levodopa intake (mean ± SD).

Groups	PD off state	PD on state	*t*	*p*-value
Total UPDRS-III	33.6 ± 13.4	16.4 ± 7.0	12.06	<0.001
Tremor	6.1 ± 4.0	2.2 ± 2.0	6.21	<0.001
Rigidity	8.1 ± 4.1	3.7 ± 2.5	10.06	<0.001
Bradykinesia	12.8 ± 5.5	5.7 ± 3.3	11.36	<0.001
Axial symptoms	4.5 ± 2.4	2.9 ± 1.2	4.70	<0.001

*UPRDS III total score and subscores between the PD off state and PD on state using paired t-tests; UPDRS-III were divided into subscores for tremor (UPDRS items 20 and 21), rigidity (UPDRS item 22), bradykinesia (UPDRS items 23–26 and 31) and axial symptoms (UPDRS items 27–30). UPDRS-III, Unified Parkinson’s Disease Rating Scale motor section*.

### fMRI Procedure

fMRI was performed using a 3T MRI scanner (Siemens, Verio, Germany). All subjects lay supine with their head fixed by foam pads with a standard birdcage head coil to minimize head movement. The participants were instructed to remain as still as possible, close their eyes, and remain awake without thinking of anything. Axial anatomical images were acquired using a T1 fluid attenuated inversion recovery sequence (repetition time [TR] = 2,530 ms; echo time [TE] = 3.34 ms; flip angle [FA] = 7 degrees; matrix = 256 × 192; field of view [FOV] = 256 mm × 256 mm; slice thickness/gap = 1.33/0.5 mm; 128 slices covered the whole brain) for image registration and functional localization. Functional images were subsequently collected in the same slice orientation with a gradient-recalled echo-planar imaging pulse sequence (TR = 2,000 ms; TE = 30 ms; FA = 90 degrees; matrix = 64 × 64, FOV = 220 mm × 220 mm; time points = 140; thickness/gap = 3.5/0.6 mm; in-plane resolution = 3.4 mm × 3.4 mm^2^; slice numbers = 31). For each subject, every rs-fMRI session lasted 280 s.

### Preprocessing and Functional Connectivity Analysis

Preprocessing of the fMRI data was carried out using the toolbox for Data Processing and Analysis for (Resting-State) Brain Imaging (DPABI 3.1^1^) based on the MATLAB 2014b platform. The first 10 volumes of functional images were discarded to allow for signal equilibrium and participant adaptation to the scanning environment. The remaining images were corrected by realignment, accounting for head motion, were normalized into the standard space using diffeomorphic anatomical registration through exponentiated Lie algebra (DARTEL), resampled to a 3 mm × 3 mm × 3 mm voxel size, had the nuisance variables regressed out, and were spatially smoothed with a 4-mm full width at half-maximum (FWHM) Gaussian kernel. The resulting fMRI data were bandpass filtered (0.01 < *f* < 0.1 Hz) before proceeding to the next step. The nuisance variables included 24 motion parameters (six head motion parameters, six head motion parameters one time point before, and the 12 corresponding squared items), the signal averaged over the individual segmented cerebrospinal fluid (CSF) and white matter (WM) regions, and the linear and quadratic trends (Yan et al., 2013b).

Previous research found that the Friston-24 covariates showed the greatest reductions in both positive and negative motion–blood oxygen-level dependent (BOLD) relationships. In addition, the Friston-24 approach produced the fewest motion-related spikes when examining the BOLD signal after head motion correction (Yan et al., 2013a). To limit the impact of head motion, we defined subjects with a mean framewise displacement (FD) of more than three interquartile ranges from the sample median as outliers and excluded them from further analysis (Zhou et al., 2018).

Twelve subregions of the M1 from the Human Brainnetome Atlas^2^ were selected for this study, including the bilateral head and face region, bilateral caudal dorsolateral area 6, bilateral upper limb region, bilateral trunk region, bilateral tongue and larynx region, and bilateral caudal ventrolateral area 6, as the ROIs. A seed reference time course was obtained within each ROI. Correlation analyses were conducted on the seed reference and the whole brain in a voxelwise manner for each ROI. The correlation coefficients of each voxel were normalized to *Z*-scores with Fisher’s *r*-to-*z* transformation. An entire brain *Z*-score map was created for each ROI for each subject.

### Statistical Analysis

For demographic statistical analysis, we used the software of SPSS 24.0 (Statistical Product and Service Solutions). The two-sample *t*-tests were applied to calculate the differences in age, education, and MMSE scores between PD patients and HCs. Group differences in sex were analyzed using the chi-squared test. For fMRI data statistical analysis, we used the statistical module of DPABI. We used the paired *t*-test to calculate the differences in FC between PD on state and PD off state for each ROI. Two-sample *t*-tests were applied to examine differences in FC between the PD off state and HC, and the PD on state and HC for each ROI. In the FC study, all results were corrected by Gaussian random field (GRF) theory (voxel-level *p* < 0.001, cluster-level *p* < 0.05) with gray matter volume as a covariate. The specific steps are as follows: All statistical parametric maps were processed using an uncorrected threshold of *p* < 0.001. Thereafter, significant clusters were detected using *p* < 0.05 with familywise error (FWE) correction at the cluster level.

A correlation analysis of ΔFC values (FC between PD off state and PD on state for the significant clusters) against changes in UPDRS-III total and subscale scores (including Δtremor, Δrigidity, Δbradykinesia, and Δaxial symptoms) was performed to explore the relationship between the changes in FC in the motor circuit and improvements in clinical symptoms. Meanwhile, disease duration and levodopa equivalent daily dose (LED) were introduced as covariates in each correlation analysis.

## Results

### Demographic and Clinical Characteristics

Scanning proceeded with no adverse effects. As shown in Table 1, there was no significant difference in sex (*p* = 0.903), age (*p* = 0.211), MMSE score (*p* = 0.112), or education years (*p* = 0.681) between PD patients and controls. After levodopa intake, the UPDRS motor score was significantly improved in all patients. The mean improvement in UPDRS-III was 51% [*SD* = 0.12; 95% CI = 0.47–0.55]. The clinical symptom tremor was reduced by 57% [*SD* = 0.35; 95% CI = 0.45–0.69], rigidity by 54% [*SD* = 0.23; 95% CI = 0.46–0.62], bradykinesia by 55% [*SD* = 0.25; 95% CI = 0.47–0.63], and axial symptoms by 28% [*SD* = 0.30; 95% CI = 0.18–0.38]. The mean LED was 455.2 mg (*SD*, 232.4 mg). The LED showed a significant correlation with ΔUPDRS-III scores (*r* = 0.421, *p* = 0.011) and Δbradykinesia scores (*r* = 0.445, *p* = 0.007) during the levodopa challenge test.

### Functional Connectivity

We mainly focused on the comparison between PD on state and PD off state to observe the effect of levodopa on the motor circuit. There are three subregions (see Table 3) that showed a significant difference in FC between these states. These three subregions also showed significantly decreased FC when compared between PD patients in the off state and HC, PD patients in the on state and HC. The three subregions of M1 and the details are as follows:

**TABLE 3:** Changes in functional connectivity between PD on and PD off states.

Seed ROI	Brain region (AAL)	L/R	BA	Peak MNI coordinates			Cluster size (voxels)	*t*-value
				
				*X*	*Y*	*Z*		
**PD on < PD off**								
A6cdl	ACC	L	24	0	18	30	37	−5.23
A4ul	mdTHA	L		−6	−21	6	21	−4.69
**PD on > PD off**								
A4tl	MFG	L	9	−9	51	18	30	5.49

*ROI, region of interest; AAL, Anatomical Automatic Labeling; L/R, left/right; BA, Brodmann area; MNI, Montreal Neurological Institute; A6cdl, caudal dorsolateral area 6; A4ul, area 4 upper limb region; A4tl, area 4 tongue and larynx region; ACC, anterior cingulate gyrus; mdTHA, medial dorsal thalamus; MFG, medial frontal gyrus. The results represent a comparison of functional connectivity in ROIs between the PD on and PD off states (GRF-corrected, voxel-level p < 0.001, cluster-level p < 0.05).*

#### Right Caudal Dorsolateral Area 6

**FIGURE 2:**
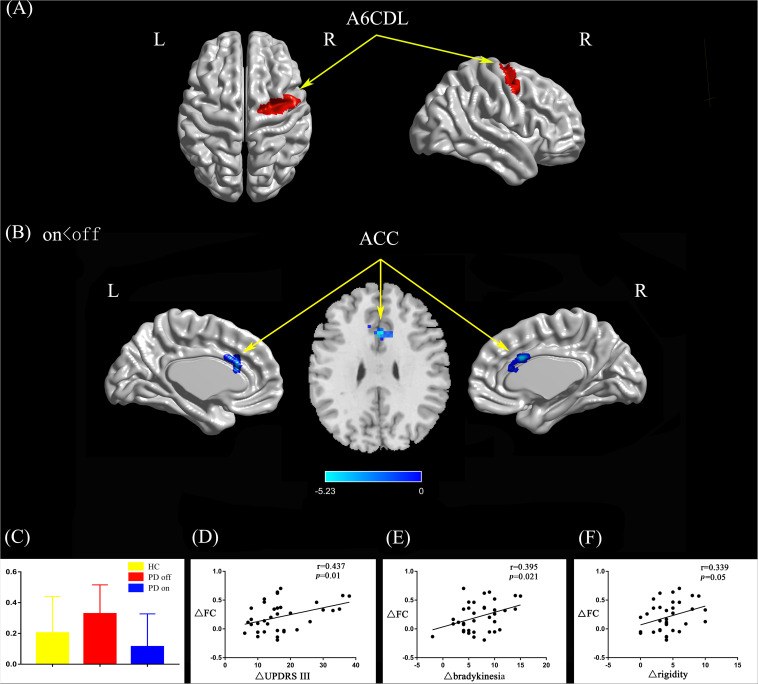
**(A)** The location of the right caudal dorsolateral area 6 (red color) based on the Brainnetome Atlas template. **(B)** Brain region (ACC) showed significant difference in functional connectivity with right caudal dorsolateral area 6 between PD on and PD off states (paired *t*-test, voxel-level *p* < 0.001, cluster-level *p* < 0.05, GRF correction), the cold color indicates decreased functional connectivity in PD on state compared with PD off state (PD on < PD off). **(C)** FC value histogram for the ACC in the three groups (HC, PD off, PD on). **(D–F)** ΔFC between the ACC and right caudal dorsolateral area 6 shows a positive correlation with ΔUPDRS-III scores **(D)**, Δbradykinesia scores **(E)**, and a trend toward a positive correlation with Δrigidity scores **(F)**. A6CDL, caudal dorsolateral area 6; ACC, anterior cingulate gyrus; ΔFC, difference in functional connectivity between PD on and off states; ΔUPDRS-III scores, improvement in symptom scores on UPDRS part III between PD on and off states; Δbradykinesia, improvement in symptom scores on UPDRS-III items 23–26 and 31; Δrigidity, improvement in symptom scores on UPDRS-III item 22; L/R, left/right.

Compared with the PD off state, the PD on state exhibited decreased connectivity between the right caudal dorsolateral area 6 and the anterior cingulate gyrus (ACC) (Figures 2A,B). We extracted the FC values and made a histogram (Figure 2C). Compared with the HC, the PD patients in the off state exhibited decreased connectivity between the right caudal dorsolateral area 6 and right middle occipital gyrus and right cuneus (Supplementary Figure S1A). Compared with the HC, the PD patients in the on state showed decreased connectivity between the right caudal dorsolateral area 6 and the bilateral cerebellum posterior lobe and cuneus (Supplementary Figure S1B).

#### Right Upper Limb Region

**FIGURE 3:**
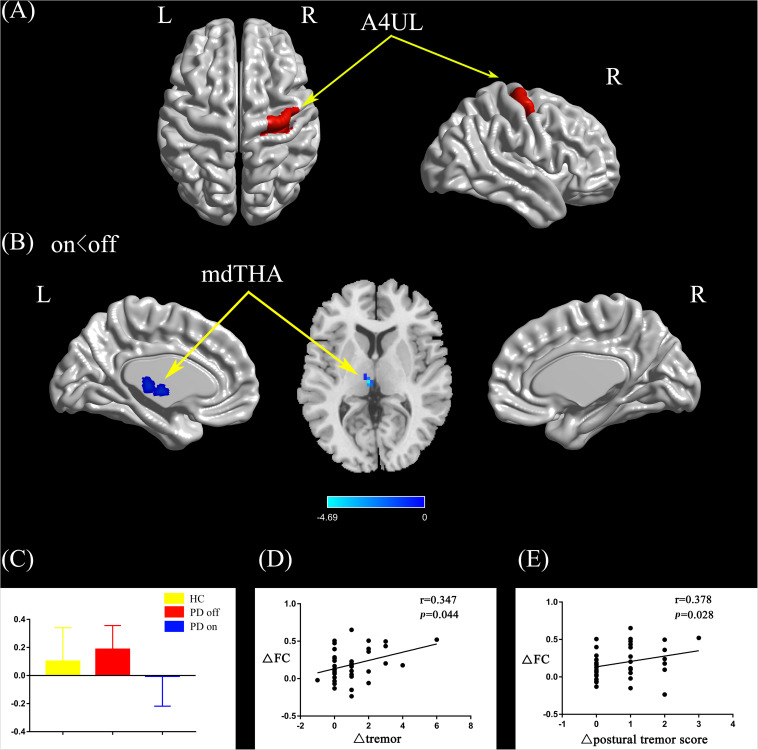
**(A)** The location of the right upper limb region (red color) based on the Brainnetome Atlas template. **(B)** Brain region (left mdTHA) showed a significant difference with the right upper limb region in functional connectivity between PD on and PD off (paired *t*-test, voxel-level *p* < 0.001, cluster-level *p* < 0.05, GRF correction), and the cold color indicates decreased functional connectivity in PD on state compared with PD off state (PD on < PD off). **(C)** FC value histogram for the left mdTHA in the three groups (HC, PD off, PD on). **(D,E)** ΔFC between the right upper limb region and the left mdTHA show a significant positive correlation with the Δtremor scores of the left upper limb **(D)** and Δpostural tremor scores of the left upper limb **(E)**. A4UL, area 4 upper limb region; mdTHA, medial dorsal thalamus; ΔFC, difference in functional connectivity between PD on and off states; Δtremor, improvement in symptom scores on UPDRS-III items 20c and 21b (left upper limb); Δpostural tremor score, improvement in symptom scores on UPDRS-III item 21b (left upper limb); L/R, left/right.

Compared with the PD off state, the PD on state exhibited decreased connectivity between the right upper limb region and the left medial dorsal thalamus (mdTHA) (Figures 3A,B). We extracted the FC values and made a histogram (Figure 3C). Compared with the HC, the PD patients in the off state exhibited decreased connectivity between the right upper limb region and the bilateral middle occipital gyrus, bilateral middle temporal gyrus, bilateral precentral gyrus, and bilateral postcentral gyrus (Supplementary Figure S1C). Compared with the HC, the PD patients in the on state showed decreased connectivity between the right upper limb and the bilateral middle occipital gyrus, bilateral cuneus, and bilateral lingual gyrus (Supplementary Figure S1D).

#### Left Tongue and Larynx Region

**FIGURE 4:**
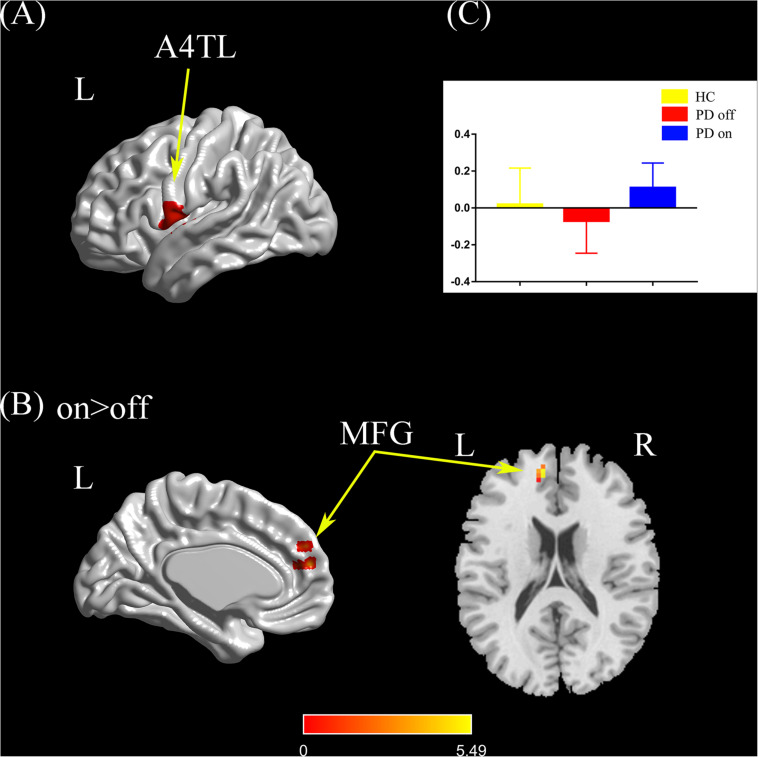
**(A)** The location of the left tongue and larynx region (red color) based on the Brainnetome Atlas template. **(B)** Brain region (left MFG) showed a significant difference with the left tongue and larynx region in functional connectivity between PD on and PD off (paired *t*-test, voxel-level *p* < 0.001, cluster-level *p* < 0.05, GRF correction), and the hot color indicates increased functional connectivity in PD on state compared with PD off state (PD on > PD off). **(C)** FC value histogram for the left MFG in the three groups (HC, PD off, PD on). A4TL, area 4 tongue and larynx region; MFG, medial frontal gyrus; L/R, left/right.

Compared with the PD off state, the PD on state exhibited increased connectivity between the left tongue and larynx region and the left medial frontal gyrus (Figures 4A,B). We extracted the FC values and made a histogram (Figure 4C). Compared with the HC, the PD patients in the off state exhibited no significant difference in connectivity with the left tongue and larynx region. Compared with the HC, the PD patients in the on state showed decreased connectivity between the left tongue and larynx region and the right middle frontal gyrus (Supplementary Figure S1E).

### Correlation Analysis

After controlling for disease duration and LED as covariates in the correlation analysis, we found that the ΔFC between the right caudal dorsolateral area 6 and ACC showed a significant positive correlation with ΔUPDRS-III scores (*r* = 0.437, *p* = 0.01) and Δbradykinesia scores (*r* = 0.395, *p* = 0.021) and a trend toward a positive correlation with Δrigidity scores (*r* = 0.339, *p* = 0.05) (Figures 2D–F). We also explored the relationships between ΔFC (between the right upper limb region and left mdTHA) and improved motor symptoms, with no significant correlations found. However, we further explored the relationship between ΔFC in the right upper limb subregion, which mainly controls movement of the contralateral limb, and motor symptoms of the left upper limb. This ΔFC (between the right upper limb region and left mdTHA) showed a significant positive correlation with the Δtremor score of the left upper limb (UPDRS-III items 20c and 21b; *r* = 0.347, *p* = 0.044) and Δpostural tremor score of the left upper limb (UPDRS-III items 21b; *r* = 0.378, *p* = 0.028) (Figures 3D,E). The ΔFC between the left tongue and larynx region and the left medial frontal cortex (MFC) showed no significant correlation with improvements in symptom scores.

## Discussion

This study explored the changes in FC patterns in different subregions of the M1 in PD patients after levodopa intervention. We found that subregions of the M1, including the right caudal dorsolateral area 6, right upper limb region, and left tongue and larynx region, showed significantly different changes in FC after levodopa drug intervention. Furthermore, the above changes in FC were associated with improvements in specific clinical symptoms. First, the decreased FC between the right caudal dorsolateral area 6 and ACC was positively correlated with improvements in bradykinesia and rigidity. Second, the decreased FC between the right upper limb region and the left mdTHA was positively related to improvements in the left upper limb tremor. Our results suggest that the subregions of the M1 have different changes in connection patterns after levodopa administration. These changes in connection patterns influenced multiple motor circuits or brain networks and subsequently correlated with improvements in clinical symptoms.

Previous studies have identified that the ACC plays an important role in the regulation of multiple brain functions, such as cognition, emotion, and motor execution, which are involved in the default mode network, limbic system, executive networks, and so on (Isomura and Takada, 2004; Goulden et al., 2014; Christopher et al., 2015; Palermo et al., 2018; Wei et al., 2018). For instance, Christopher et al. (2015) found in a PET study that PD patients with mild cognitive impairment (MCI) demonstrated more significant reductions in D2 receptor binding in the ACC than HC and patients with no MCI. In our research group, Wei et al. (2018) found that depressed PD (DPD) patients exhibited increased FC between the ACC and ventral tegmental area (VTA) relative to HC and non-depressed PD (NDPD) patients. Meanwhile, aberrant FC was correlated with the severity of depression in PD patients (Wei et al., 2018).

However, although the ACC is involved in the regulation of the above brain networks, those networks are mainly related to the non-motor symptoms of PD, such as depression and cognitive impairment, which are considered to rarely respond to levodopa. Instead, levodopa has a much stronger effect on motor symptoms than non-motor symptoms (Martinu et al., 2012). It is well known that levodopa acts on the classic BGMC circuit, balancing the direct and indirect pathways at the striatal level, increasing the excitatory outflow to the MC and thus improving the motor symptoms associated with PD (Gao et al., 2017; Manza et al., 2018). More importantly, DeLong and Wichmann (2015) proposed that the motor circuit originates and terminates in cortical precentral areas, including the M1, SMA, premotor cortex (PMC), and cingulate motor area (CMA) (Poston and Eidelberg, 2012; DeLong and Wichmann, 2015). A large number of previous studies have mentioned that the CMA is located in the Brodmann 24/32 area, which is partly consistent with our location of the ACC (Francis et al., 2009; Feng et al., 2015; Wang et al., 2015; Pievani et al., 2017). Accordingly, we speculate that FC between the right caudal dorsolateral area 6 and ACC (mainly in the motor area) is directly affected by levodopa through the classic BGMC circuit, which also explains why the altered FC is related to improvements in motor symptoms that include bradykinesia and rigidity.

Currently, the relationship between motor symptoms and non-motor symptoms in PD patients remains unclear. Based on the parallel circuit model, the motor, cognitive, and limbic function circuits are segregated from each other in the cortico-basal ganglia-thalamo-cortical loop (McGregor and Nelson, 2019). This is due to a subregion of the striatum receiving glutamatergic and dopaminergic innervation from different input regions, and the outputs from these subregions to the downstream basal ganglia nuclei tend to be separated (Alexander et al., 1986). Notably, Frank (2011) found that motor and cognitive corticostriatal circuits are not completely segregated, which means that the information in the motor circuit may crosstalk with other channels at the level of the cortex when it re-enters the basal ganglia loop (Frank, 2011). In fact, this interaction has previously been simulated through neural models, and the representation of particular cognitive rules in the prefrontal cortex can directly guide the movement selection of the striatum before procedural learning occurs (Doll et al., 2009). Therefore, we speculate that the interaction of information from different corticostriatal circuits may partly occur in the ACC. Future studies are needed to further investigate the underlying mechanisms within the ACC, which may be the core node involved in both the motor and non-motor symptom-related networks in PD patients.

To our knowledge, previous studies have considered that the classic BGMC model could better explain the mechanisms of bradykinesia and rigidity than those of resting tremor (Pirker, 2003; Helmich et al., 2011; DeLong and Wichmann, 2015; Zhang et al., 2016). Tremor is considered to have a different pathological mechanism than hypokinesia in PD patients. In fact, Helmich et al. (2011) proposed that the resting tremor circuit model consisted of a VIM-MC-cerebellum (CBLM) circuit, which was connected with the BGMC circuit through the ventralis oralis posterior nucleus of the thalamus (VOP) (Helmich et al., 2011). He emphasized that resting tremor is associated with increased activity in the VIM-MC-CBLM circuit, which develops through dopaminergic dysfunction in the basal ganglia. Thus, in our research, acute levodopa intervention transiently reversed this dysfunction and significantly improved the clinical symptoms of tremor. In addition, the decreased FC between the right upper limb region and the left mdTHA was significantly positively related to improvements in the left upper limb tremor. This finding is consistent with a previous study that suggested that dopaminergic medications could specifically reduce tremor-related oscillatory coupling between the thalamus and MC in PD patients (Pollok et al., 2009). Moreover, a large number of studies have reported that the VIM of the thalamus is closely related to tremor and that DBS of the VIM could significantly improve the symptoms of tremor (Lenz et al., 1994; Klein et al., 2012; Mao et al., 2019). We found that mdTHA partly overlaps with VIM, which means that mdTHA is partly involved in the tremor circuit model.

In particular, we found that the decreased FC between the right upper limb region and the left mdTHA was significantly positively correlated with improvements in postural tremor in the left upper limb but not in resting tremor. Although parkinsonian tremors usually occur at rest, nearly 46–92% of patients have postural tremors (Koller et al., 1989; Gigante et al., 2015). In fact, postural tremor may be the first manifestation of PD and may be more prominent and disabling than resting tremor (Jankovic et al., 1999). Previous studies proposed that VIM thalamotomy or VIM DBS significantly improves all types of tremor, including resting tremor and postural tremor (Hallett and Deuschl, 2010). In addition, other studies have found that M1 stimulation could reset both postural and resting tremors, which means that M1 is involved in the generation or transmission of the above two types of tremors (Britton et al., 1993; Pascual-Leone et al., 1994; Ni et al., 2010). Consequently, VIM and M1 involvement seems to be an intrinsic feature in the two types of tremor. However, postural tremor is still considered to have a different pathological mechanism from resting tremor, and the exact phenomenology and etiology of postural tremor remain unclear (Helmich et al., 2012).

Dirkx et al. (2018) proposed that voluntary movement during posturing increases neural excitability within the cerebello-thalamo-cortical motor circuit, resulting in faster synaptic transmission and higher tremor frequency (Dirkx et al., 2018). Our results showed that FC between the right upper limb region and the left mdTHA was higher in the PD patients in the off state than in HC, which can be quickly reversed after taking levodopa (the FC becomes even lower than in HC). More importantly, the decreased FC was significantly correlated with improvements in postural tremor in the left upper limb in PD patients, which means that levodopa could normalize or restore the aberrant FC within the cerebello-thalamo-cortical motor circuit and improve the clinical symptoms of postural tremor.

According to our results, the third altered subregion in M1 was the left tongue and larynx region, which exhibited increased FC with the left medial frontal gyrus. In previous studies, the posterior medial frontal cortex (pMFC) was suggested to play an important role in movement initiation, and subdural stimulation of this region has been reported to trigger motor responses (Deiber et al., 1999; Cunnington et al., 2002; Herz et al., 2014). Another study showed that dopaminergic medication led to increased FC of pMFC, which correlated with improvements in motor performance (Michely et al., 2015). In our study, the ΔFC between the left tongue and larynx region and the ipsilateral MFC showed no significant correlation with improvements in symptom scores. This may be partly because of the pronunciation, masticatory, and swallowing function of laryngeal muscles, which is mainly controlled by the bilateral MC, and these symptoms typically occur later in the disease course. Moreover, motor dysfunction of the larynx and tongue in PD patients generally shows a poor response to levodopa treatment.

We also compared the above three subregions of the M1 between PD patients and age-, sex-, and MMSE score-matched HCs. The three subregions all showed decreased FC with several brain regions, such as the bilateral cerebellum, bilateral middle temporal gyrus, bilateral occipital gyrus, bilateral precentral gyrus, and bilateral postcentral gyrus (see Supplementary Figure S1). The above results were consistent with previous task-based fMRI studies that found reduced functional activity in M1 in both drug-naïve PD and PD off state patients (Spraker et al., 2010; Prodoehl et al., 2013; Mohl et al., 2017). Reduced functional activity in M1, partly because of the dysfunction of the basal ganglia-thalamocortical circuit, leads to a decreased excitatory outflow to the cortical motor areas (Alexander et al., 1990). Moreover, we presume that after long-term chronic degeneration, the decreased excitability of the M1 in advanced PD patients is difficult to completely reverse with levodopa administration.

There are some limitations in the present study. First, the PD patients we enrolled were mostly above Hoehn and Yahr stage 2, which means that the patients had a longer disease duration. Although these patients have typical clinical symptoms and good responsiveness to levodopa, the changes in the motor circuit we observed tended to be associated with the middle and advanced stages of the disease. Early PD patients also have good responsiveness to levodopa, and the underlying mechanisms of the altered connection patterns in motor circuits deserve further exploration. Second, this study revealed M1 subregion changes only under acute levodopa intervention. However, it is unclear how the M1 subregions change under the long-term effects of levodopa intervention. This needs to be explored in future experiments. Third, the statistical correction method we used is a cluster-defining-threshold (CDT) approach, which might import potential false positive rates. However, we set the voxel-level *p* < 0.001 and cluster-level *p* < 0.05 as the previous studies used (Eklund et al., 2016; Lin et al., 2019; Yang et al., 2019; Wang et al., 2020; Wu et al., 2020) to reduce the false positive rate as much as possible. Future studies are required to verify the results with a larger sample size and different statistical correction methods. Finally, we focused on improvements in motor symptoms rather than non-motor symptoms after acute levodopa intervention. In particular, the recruited PD patients needed a second MRI scanning and UPDRS-III score assessment when the drug achieved the best effect, with barely enough time to complete the non-motor symptom scale, such as the Montreal Cognitive Assessment (MoCA), MMSE, and Hamilton Depression Rating Scale (HAMD). In addition, the appropriate measurements that could identify and quantify immediate improvements in non-motor symptoms have rarely been reported in similar studies. Therefore, we will focus on addressing the above limitations in the future.

## Conclusion

The present study demonstrated that following levodopa drug intervention, subregions of the M1 showed different changes in connection patterns, different motor circuits were influenced, and the specific clinical symptoms of PD were improved. Specifically, the right caudal dorsolateral area 6 and ACC may participate in the classic BGMC circuit and improve the symptoms of bradykinesia and rigidity. The right upper limb region and the left mdTHA were involved in the tremor circuit and improved the postural tremor of the left upper limb. In addition, the ACC is associated with multiple clinical symptoms of PD, which may further allow us to probe the relationship between motor and non-motor symptoms. Our results reveal novel information regarding the underlying mechanisms in the motor circuits in the M1 and a promising way to explore the internal function of the M1 in PD patients. Notably, M1 is a potential therapeutic target in PD, and the exploration of its subregion provides a basis and new insights for clinical intervention and precise drug treatment.

## Data Availability Statement

The datasets generated for this study are available on request to the corresponding author.

## Ethics Statement

The studies involving human participants were reviewed and approved by Medical Ethics Committee of the Affiliated Brain Hospital of Nanjing Medical University. The patients/participants provided their written informed consent to participate in this study.

## Author Contributions

WeL and MY designed and organized the research. YS, JH, YL, and LY collected the imaging and assessment scale data. YS and WaL analyzed the data and wrote the manuscript. MY, YC, and CX made important revisions to the manuscript. WeL and WZ approved the final version of the manuscript to be published. All authors contributed to the article and approved the submitted version.

## Conflict of Interest

The authors declare that the research was conducted in the absence of any commercial or financial relationships that could be construed as a potential conflict of interest.

## Footnotes

1 http://rfmri.org/dpabi

2 http://atlas.brainnetome.org

## Supplementary Material

FIGURE S1 Brain regions showed significant differences in functional connectivity with right caudal dorsolateral area 6 between PD off state and HC **(A)** and between PD on state and HC **(B)**. Brain regions showed significant differences in functional connectivity with right upper limb region between PD off state and HC **(C)** and between PD on state and HC **(D)**. Brain region showed significant difference in functional connectivity with left tongue and larynx region between PD on state and HC **(E)**.
