# Supplementary figures and images for: Levodopa Changes Functional Connectivity Patterns in Subregions of the Primary Motor Cortex in Patients With Parkinson’s Disease
Source: Front Neurosci. 2020 Jul 8;14:647. doi: 10.3389/fnins.2020.00647 (PMC7360730; doi:10.3389/fnins.2020.00647)

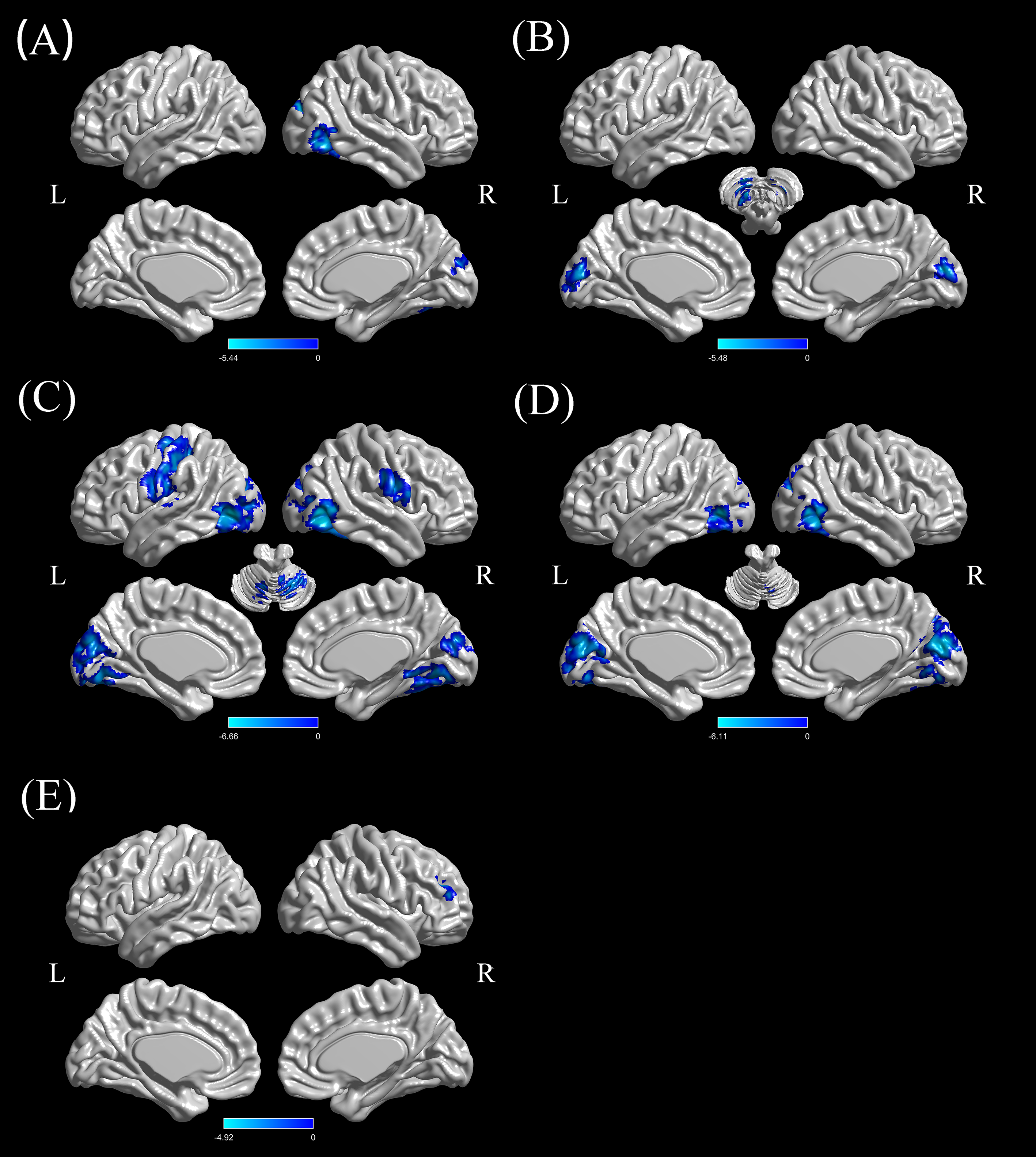

Supplement: FIGURE S1 — Brain regions showed significant differences in functional connectivity with right caudal dorsolateral area 6 between PD off state and HC (A) and between PD on state and HC (B). Brain regions showed significant differences in functional connectivity with right upper limb region between PD off state and HC (C) and between PD on state and HC (D). Brain region showed significant difference in functional connectivity with left tongue and larynx region between PD on state and HC (E). [file Image_1.TIF]
