# Supplementary material for: Levodopa Changes Functional Connectivity Patterns in Subregions of the Primary Motor Cortex in Patients With Parkinson’s Disease
Source: Front Neurosci. 2020 Jul 8;14:647. doi: 10.3389/fnins.2020.00647 (PMC7360730; doi:10.3389/fnins.2020.00647)
Supplement: Supplementary file 2 [file Table_1.DOC]

Supplementary table Brain regions showed significant differences in functional connectivity with A6cdl, A4ul and A4tl between PD on state and HC, PD off state and HC.

| Seed-ROI | Brain Region（AAL） | L/R | BA | Peak-MNI coordinate | | | Clusters size  (voxels) | T Value |
| --- | --- | --- | --- | --- | --- | --- | --- | --- |
|  |  |  |  | X | Y | Z |  |  |
| **PD on < HC** |  |  |  |  |  |  |  |  |
| A6cdl | Cerebellum Posterior Lobe | R |  | 12 | -69 | -54 | 70 | -5.48 |
|  | Cerebellum Posterior Lobe | L |  | -21 | -60 | -57 | 113 | -5.08 |
|  | Cuneus | L | 18 | -6 | -84 | 12 | 98 | -4.50 |
| A4ul | Middle Occipital Gyrus | R |  | 48 | -66 | -3 | 187 | -6.11 |
|  | Cuneus | L |  | -45 | -72 | 0 | 698 | -5.54 |
|  | Lingual | R |  | 12 | -84 | -9 | 49 | -4.75 |
| A4tl | Middle Frontal Gyrus | R | 10 | 36 | 45 | 27 | 57 | -4.92 |
| **PD off < HC** |  |  |  |  |  |  |  |  |
| A6cdl | Middle Occipital Gyrus | R |  | 48 | -69 | -3 | 118 | -5.44 |
|  | Cuneus | R |  | 18 | -90 | 24 | 69 | -4.87 |
| A4ul | Middle Occipital Gyrus  Middle Temporal Gyrus | R | 18/19 | 45 | -69 | -3 | 1573 | -6.66 |
|  | Precentral Gyrus  Postcentral Gyrus | L | 4/6 | -45 | 18 | 39 | 391 | -5.77 |
|  | Precentral Gyrus  Postcentral Gyrus | R | 4/6 | 51 | -9 | 21 | 164 | -5.68 |
| A4tl | NONE |  |  |  |  |  |  |  |

Abbreviations: BA, Brodmann area; ROI,region of interest; AAL,Anatomical Automatic Labeling; MNI, Montreal Neurological Institute; A6cdl, caudal dorsolateral area 6; A4ul, area 4 upper limb region; A4tl, area 4 tongue and larynx region; Results are the comparison of functional connectivity in ROIs between the PD on and HC, PD off and HC (GRF-corrected, voxel-level *p* <0.001, cluster-level *p* <0.05).
